# Re-pericardiectomy for recurrent chronic constrictive pericarditis: left anterolateral thoracotomy is a better approach

**DOI:** 10.1186/s13019-019-0978-8

**Published:** 2019-08-22

**Authors:** Ling Yunfei, Li Tao, Qian Yongjun

**Affiliations:** Department of Cardiovascular Surgery, West China Hospital, Sichuan University, Guoxuexiang 37th, 610041 Chengdu, Sichuan People’s Republic of China

**Keywords:** Constrictive pericarditis, Pericardiectomy, Reoperation, Left anterolateral thoracotomy

## Abstract

**Background:**

Pericardiectomy is the final treatment for constrictive pericarditis. However, this greatest surgical approach is still very controversial. This study pursued to assess the outcomes in patients with recurrent chronic constrictive pericarditis undergoing reoperated pericardiectomy via median sternotomy versus left anterolateral thoracotomy and to explain which surgical approaches might be better for recurrent chronic constrictive pericarditis.

**Methods:**

A total of 24 patients were identified with recurrent chronic constrictive pericarditis and underwent reoperation with pericardiectomy between July 2003 and July 2015. The decision for this surgical approach was mainly dependent on the operating surgeon’s preference. Out of 20 patients, 16 patients underwent pericardiectomy via median sternotomy and 8 patients via left anterolateral thoracotomy pericardiectomy. Their data were obtained retrospectively from the case notes.

**Results:**

Both groups of patients were similar in age, gender between two operations, and also in peripheral venous pressure, cardiac rhythm and New York Heart Association (NYHA) class distribution. The mortality rates were similar in both groups with one death (12.5%) due to low cardiac output syndrome in the left anterolateral thoracotomy group and two deaths (12.5%) in the median sternotomy group. All the deaths were associated with cardiac complications and happened in the perioperative period. NYHA functional class status enhanced in most of the patients. Patients in both groups had a similar and significant improvement in their NYHA status that improved from 3.4 ± 0.7 to 1.8 ± 0.1 (*P* = 0.001) in the left anterolateral thoracotomy group and reduced from 3.3 ± 0.6 to 1.9 ± 0.4 (*P* = 0.001) in the median sternotomy group. There was a significantly greater rate of pulmonary infection in the thoracotomy group than in the median sternotomy group (50% versus 25%, *P* = 0.02). Nevertheless, there was a significantly greater occurrence of wound infections in the median sternotomy group in 3 patients versus in one patient of the left anterolateral thoracotomy group (18.8% versus 12.5%, *P* = 0.02).

**Conclusions:**

Left thoracotomy incision was preferred to sternotomy in the current setting of this situation and was done safely without CPB. It avoided life-threatening sternal infection and it also has showed an equal as well las significant enhancement of NYHA status of the patients.

## Background

Chronic constrictive pericarditis is a disease which is characterized by noticeable thickening as well as thick scarring of the pericardium with pericardial sac obliteration, or calcification of the pericardium. Surgical pericardiectomy is extremely effective and possibly restorative for the heart failure [1]. However, some patients still have recurrent pericarditis. Reoperated pericardiectomy has been established as a potential and promising substitute to supplementary medical treatments in patients with refractory recurrent pericarditis [2].

The different approaches available for pericardiectomy are median sternotomy, left anterolateral thoracotomy and bilateral anterior thoracotomy; and the selection of one of these approaches is based on the personal preferences [3–5]. It has been also reported that pericardiectomy improves NYHA status in all patients and mortality rates are parallel among the above mentioned approaches [6]. Now the question is that the same approach lead to the requirement of surgeries in recurrent chronic constrictive pericarditis patients. Even though, there are a lot of decent outcomes authenticating the advantages of pericardiectomy by means of diverse surgical approaches as mentioned in individual case reports [7, 8]. So far there has not been any specific study which has addressed the choice of a specific surgical approach in the setting of recurrent pericarditis, which comorbid conditions contribute most to postoperative morbidity as well as mortality. Moreover, the long-term outcomes after intervention are not reported either which are very important to understand the appropriate effects of the intervention.

Here, in the current study, the main aim was to evaluate the outcomes in patients with recurrent chronic constrictive pericarditis undergoing reoperated pericardiectomy via median sternotomy versus left anterolateral thoracotomy. We also aimed to analyze the clinical characteristics and to compare the consequences of morbidity and mortality. One of our goals were to explain which surgical approaches might be better for recurrent chronic constrictive pericarditis.

## Patients and methods

### Patient selection

Recurrence or reappearance was defined as the consequent incidence of a symptomatic pericardial constrictive after first pericardial procedure. The term chronic is usually referred, particularly for constrictive pericarditis, in which the disease processes last for > 3 months [9].

All patients with the diagnosis of recurrent tuberculous chronic constrictive pericarditis who underwent reoperated pericardiectomy between July 2003 and July 2015 at three different hospitals were included in the current study. Diagnosis was done by performing echocardiography on all patients. X-ray films and computed tomography (CT) or magnetic resonance imaging (MRI) scans of the chests revealed protuberant calcification of the pericardium as well as thickness of more than 3 mm. Peripheral venous pressures were also increased. During the above mentioned period, 24 patients were identified and their data were collected retrospectively from the case notes. The first scheduled pericardiectomy was done by median sternotomy in all patients. The patients who required other surgeries (such as valve replacement or coronary artery bypass surgery) were excluded from the study. In formed consents were obtained from all patients for their participation in this study. The experimental protocol was approved by the hospital’s ethics committee.

The decision for the approach was dependent on the operating surgeon’s preference. All the operations were performed by three surgeons. Details regarding follow-up were obtained from the clinic visits, mailed questionnaires to patients or families, as well as over the phone until July 2016. All the patients were evaluated at week 4 and week 24 after the surgery and their clinical status was assessed and any complications following the discharge were documented. If the deaths occurred during the 4 weeks’ follow-up period whether before or after discharge were included in operative mortality.

### Surgical technique

In the current study, we reviewed 24 cases of pericardiectomy which were mainly carried out for recurrent chronic constrictive pericarditis. We compared the outcomes of pericardiectomy achieved by left anterolateral thoracotomy versus median sternotomy without cardiopulmonary bypass (CPB). All patients were operated under general anesthesia along with endotracheal intubation. All patients had complete cardiovascular monitoring comprising invasive blood pressure as well as central venous pressure monitoring.

Pericardiectomy was accomplished via median sternotomy in 16 patients. The resection over the left ventricle extended to the left phrenic nerve whenever possible. The pericardium was decorticated in the following order: first from the aorta and pulmonary artery, including the left ventricular outflow tract, then from the left and right ventricles and left and right atrium, and lastly the superior, inferior venae cava and diaphragm were carried out whenever possible. The resection of pericardium over the atria and the major vessels was done depending on the acceptability of the exposure and the probability of obtaining the cleavage plane. CPB was kept on standby.

The left anterolateral thoracotomy pericardiectomy was done in 8 patients through the fourth intercostal space by means of a chest wall retractor. Following thoracotomy, the pericardium over the left and partial right ventricles was removed in all cases. The resection over the left ventricle extended to the left phrenic nerve in all cases whenever possible. To avoid injury to phrenic nerves, the entire anterior pericardium was decorticated within 2-3 cm of the phrenic nerves. CPB was also kept on standby.

A drain (Nu. 28F) was inserted by making a discrete incision. The drain was detached once the daily drainage was below 100 ml. All collected specimens were sent for histological and microbiological analyses.

### Statistical analysis

Continuous data were expressed as mean ± standard deviation, which included preoperative left ventricular ejection fraction, age at the operation, cardiopulmonary bypass time, and duration of follow-up. Variables were not normally distributed and non-parametric analyses were performed. Data between 2 groups were compared using Fisher exact test and chi-square test for categorical variables. And a *P* value of less than 0.05 was considered to show statistically significant difference.

## Results

### Preoperative data

Pericardiectomy was done by median sternotomy in 16 patients and by left anterolateral thoracotomy in 8 patients. Preoperative clinical characteristics of 24 patients with recurrent constrictive pericarditis in the study cohort are mentioned in Table 1. Mean age at operation was 31.4 (for patients undergoing median sternotomy) and 33.5 (for patients undergoing left anterolateral thoracotomy) (range, 14–61 years). Other preoperative comorbid conditions are presented in Table 1. Patients in both groups were similar in age, sex distribution, interval between of twice operations, peripheral venous pressure, cardiac rhythm and New York Heart Association (NYHA) class. Seen in Table 1.

**Table 1:** Preoperative clinical characteristics of 24 patients with recurrent constrictive pericarditis in the study cohort

	Median sternotomy (*n* = 16)	Left anterolateral thoracotomy (*n* = 8)	P
Age (years)	31.4 ± 8.6	28.9 ± 8.8	0.76
Sex(M:F)	9:7	5:3	0.53
Interval between two operations (years)	6.4 ± 3.1	5.3 ± 4.1	0.42
peripheral venous pressure (cmH_2_O)	30.4 ± 9.2	28.9 ± 8.8	0.82
Cardiac rhythm (SR:AF)	9:7	4:4	0.88
NYHA			
II(%)	3	1	0.25
III (%)	6	3	0.37
IV(%)	7	4	0.41
LVEF (%)	53.5 ± 10.7	52.8 ± 7.6	0.65

Abbreviations: *LVEF* left ventricular ejection fraction, *SR* Sinus Rhythm, *AF* Atrial Fibrillation, *NYHA* New York Heart Association, *M* Males, *F* Females

### The results of postoperative complications and functional status by different surgical approaches

We found that the mortality rates were similar in both groups with one death (12.5%) as a result of low cardiac output syndrome in the left anterolateral thoracotomy group and two deaths (12.5%) in the median sternotomy group. All the deaths were cardiac related and happened during the perioperative period. In median sternotomy group, one death was due to sternal dehiscence and multisystem organ failure and the other death was due to low cardiac output. In the left anterolateral thoracotomy group, one reoperated patient was reported to have calcifications into myocardium on left side, even though we preferred to use left anterolateral thoracotomy. However, the left ventricular ejection fraction did not show any improvement and 1 week later the patient died in hospital due to low cardiac output.

In the median sternotomy group, the mean peripheral venous pressures reduced from 31.4 ± 8.6cmH_2_O to 16.4 ± 7.6 cmH_2_O (*P* < 0.01). In the left anterolateral thoracotomy group, the mean peripheral venous pressures reduced from 28.9 ± 8.8 cmH_2_O to 17.5 ± 5.6 cmH_2_O (*P* < 0.01). These findings suggest that both groups had a similar and significant improvement in their mean peripheral venous pressures (Table 2).

**Table 2:** Postoperative complications and functional status by different surgical approaches

	Median sternotomy (*n* = 16)	Left anterolateral thoracotomy (*n* = 8)	P
Mortality (%)	2(12.5)	1(12.5)	0.81
peripheral venous pressure (cmH_2_O)	16.4 ± 7.6	17.5 ± 5.6	0.57
Cardiac rhythm (SR:AF)	9:7	6:2	0.02
Operation time (min)	110.3 ± 50.6	96.2 ± 30.1	0.01
Wound infection (%)	3(18.8)	1(12.5)	0.02
Pulmonary complications (%)	4(25)	4(50)	0.01
Postoperative hospital stay	9.5 ± 8.0	8.8 ± 5.0	0.2
NYHA			
I (%)	7	4	0.41
II(%)	7	2	0.18
III (%)	2	1	0.37
IV(%)	1	1	0.82
LVEF (%)	55.2 ± 11.4	53.7 ± 10.1	0.56

Abbreviations: *LVEF* left ventricular ejection fraction, *SR* Sinus Rhythm, *AF* Atrial Fibrillation, *NYHA* New York Heart Association

However, a significant difference was observed in the duration of the operation time. The operation to perform left anterolateral thoracotomy was finished easily and early compare to the median sternotomy group. Thus, left anterolateral thoracotomy had reduced the operation time (96.2 ± 30.1vs. 110.3 ± 50.6, *P* = 0.01). The cardiac failure symptoms were gradually relieved after one to 2 weeks after pericardiectomy. After the surgery, follow-up was done for all patients and the mean duration of the follow-up was 42.2 ± 10.5 months. All patients’ main symptoms such as dyspnea, abdominal distension disappeared. However, one patient from left anterolateral thoracotomy group had moderate pitting pedal edema after 3 months. He was not able to visit the hospital regularly to adjust his diuretics medicines. Hence, when we observed him during his next follow-up, the pedal edema was obviously alleviated.

Along with similar requirement of the inotropic support during perioperative period, both groups had similar duration of hospital stay after operation.

We also observed that the NYHA functional class status was improved in most of the patients. Patients from both groups showed similar and significant improvement in their NYHA status which improved from 3.4 ± 0.7 to 1.8 ± 0.1 (*P* = 0.001) in the left anterolateral thoracotomy group and reduced from 3.3 ± 0.6 to 1.9 ± 0.4 (*P* = 0.001) in the median sternotomy group. Even though both groups had significant improvement in their functional condition, the extent of improvement was not significantly different between the two groups (*P* = 0.48). There were not any phrenic nerve lesions in all patients (Table 2).

There was a significantly greater rate of pulmonary infection in the thoracotomy group than in the median sternotomy group (50% versus 25%, *P* = 0.02). Nevertheless, there was a significantly greater incidence of wound infections in the median sternotomy group in 3 patients versus in one patient of the left anterolateral thoracotomy group (18.8% versus 12.5%, *P* = 0.02). Certain infections required treatment with antibiotic for long duration. In median sternotomy group, 2 out of the 3 patients with serious sternal dehiscence required additional operation. After debridement and sternal fixation, one patient died due to wound infection.

## Discussion

Suitable selection of the best available approach is a central part of any pericardiectomy. The choice among median sternotomy, left anterolateral thoracotomy and bilateral anterior thoracotomy seem to be individual preference for the first pericardiectomy [3–5]. The two surgical approaches (median sternotomy or left thoracotomy) were not related to the differences in overall survival for the first pericardiectomy [1]. Till today there has not been any specific study which has addresses these approaches in the setting of recurrent constrictive pericarditis after first median sternotomy pericardiectomy. As per our knowledge, this is the first retrospective study which has investigated re-pericardiectomy for recurrent chronic constrictive pericarditis via left anterolateral thoracotomy as well as median sternotomy.

Extensive pericardiectomy is the utmost imperative point for constrictive pericarditis for both first timed operated and reoperated patients. A complete pericardiectomy removes not only the anterior pericardium, but also the inferior (diaphragmatic) and left lateral pericardia (posterior to the left phrenic nerve) [10]. There is a different degree of pericardiectomy between left anterolateral thoracotomy and median sternotomy. Median sternotomy offers more radical exposure of the heart, predominantly the diaphragmatic surface; hence, most surgeons prefer to use this approach to first and redo pericardiectomy. Access to the right ventricle, right atrium, and venae cava is difficult with left anterolateral approach. Even though constrictive effect of constrictive pericarditis affects all four cardiac chambers as well as the intrapericardial portion of the cava and pulmonary veins; from a physiological outlook, the retrieval of the heart through augmented cardiac output is mainly dependent on the actions of the left ventricular chamber [3]. It has been reported that median sternotomy allows access to 26% of the left ventricular pericardium, from left anterolateral to the total area of left ventricular pericardium was 37%; hence, the left anterolateral approach permits greater access to the left ventricle [11]. The first timed pericardiectomy was done mainly by means of median sternotomy incision; however, the bad exposure of the left ventricle was the main reason which caused recurrent pericarditis. Thus the decortication of surface of the left ventricle is more essential than the first timed pericardiectomy. Although, the left anterolateral thoracotomy offers outstanding exposure of the anterolateral and inferior aspects of the left ventricle with negligible manipulation as well as retraction of the heart.

The physiological effect is established by a modification in the volume elasticity of both left and right ventricles in chronic constrictive pericarditis. Both atria of the heart have little hemodynamic advantage. It has been also reported that the median sternotomy approach was the preferred choice in the clinical subset of patients with recurrent constrictive pericarditis after partial pericardiectomy [3]. Viola recommended that resection of the pericardium overlying the right atrium and the great veins is not vital [12]. The main aim of the surgery is to release the ventricles from the constricting envelope of fibrous tissue tremendously, especially the left ventricle. It is very important to release the left ventricle to regress the symptoms rapidly and augment thecardiac output through fast enhancement of left ventricular perfusion. This is the key factor that helped us to get the good operative results for re-pericardiectomy via left anterolateral thoracotomy. Even though the left anterolateral approach was certainly less necessary when the right atrium and venae cava were comprehensively involved, both groups had a similar and significant improvement in their mean peripheral venous pressures.

It is important to note that in 2 cases of patients in left anterolateral approach group, the arrhythmia of atrial fibrillation was converted to sinus rhythm without any sort of medicine or radio frequency intervention; and the main reasons behind this might be complete left ventricular pericardiectomy and better improvement of left ventricular perfusion. Even though, the change of rhythm alleviated symptoms, it did not have any impact on the total NYHA status.

Intraoperative bleeding is a deadly intraoperative complication for re-pericardiectomy patients. When compared with the left anterolateral approach, in the events of any accidental excessive bleeding, the patients would be connected to CPB via the median sternotomy incision [13]. However, the situation would be different for reoperated patients. We should first take care of the adhesion between mediastinum and pericardium caused by first timed pericardiectomy via median sternotomy, which would augment inadvertent extreme bleeding risk throughout open sternotomy access to the mediastinum again. The use of left anterolateral approach can avoid such risk when chest is open. Fatal bleeding has been reported but is not come across usually in left anterolateral approach patients. If hemorrhage does occur, we would have to rely on the processing predetermined plan. For example, groins surgically equipped and draped, ready femoral artery venous cannula and blood cell saver; so that femoro-femoral bypass can be instituted, if required. Therefore, we assumed that it is an equivalent prospect to keep CPB on standby for patients from both groups. However, open sternotomy increases the risk of bleeding. Furthermore, the both atria have little hemodynamic advantage and surgical efforts to decorticate them may lead to severe bleeding. In the current study we did not use CPB for any of the patients.

Even though the thoracotomy patients have greater rate of pulmonary infection, it is recommended to avoid serious sternal dehiscence wound infection caused by median sternotomy. Serious sternal dehiscence is a life-threatening sternal infection that requires reoperated treatment, increases discomfort and pain in patients along with increased medical costs. Moreover, left anterolateral thoracotomy shortens the operation time significantly. Left anterolateral thoracotomy should be recommended for recurrent chronic constrictive pericarditis to avoid any life-threatening sternal infection.

## Conclusions

In conclusion, re-pericardiectomy for recurrent chronic constrictive pericarditis is a much-debated topic and hence lacks standardization. We do not consider either of the approaches ideal for re-pericardiectomy for recurrent chronic constrictive pericarditis. Left thoracotomy incision is preferred to sternotomy in the setting of this situation and was done safely without CPB. It also avoided any life-threatening sternal infection and has a similar and significant improvement in their NYHA status.

## Data Availability

Datasets used or analysed during the current study are available from the corresponding author on reasonable request.

## Abbreviations

AF: Atrial Fibrillation
F: Females
LVEF: left ventricular ejection fraction
M: Males
NYHA: New York Heart Association
SR: Sinus Rhythm
